# DNMT3B-mediated FAM111B methylation promotes papillary thyroid tumor glycolysis, growth and metastasis

**DOI:** 10.7150/ijbs.72397

**Published:** 2022-07-04

**Authors:** Xiang Zhu, Chunyuan Xue, Xiaofeng Kang, Xiaomeng Jia, Lin Wang, Muhsin H. Younis, Donghui Liu, Nan Huo, Yuchen Han, Zhao Chen, Jing Fu, Chunyu Zhou, Xiaoxiang Yao, Yimeng Du, Weibo Cai, Lei Kang, Zhaohui Lyu

**Affiliations:** 1 Department of Endocrinology, the First Medical Center of PLA General Hospital, Beijing, China.; 2 Department of Genetic Engineering, Beijing Institute of Biotechnology, Beijing, China.; 3 Departments of Radiology and Medical Physics, University of Wisconsin-Madison, Madison, WI, USA.; 4 School of Traditional Chinese Medicine, Beijing University of Chinese Medicine, Beijing, China.; 5 Department of Nuclear Medicine, Peking University First Hospital, Beijing, China.; 6 Department of Pathology, Beijing Haidian Hospital, Beijing, China.

**Keywords:** Papillary thyroid cancer, FAM111B, DNMT3B, Glycolysis, Estrogen

## Abstract

Over the past decades, the incidence of thyroid cancer (TC) rapidly increased all over the world, with the papillary thyroid cancer (PTC) accounting for the vast majority of TC cases. It is crucial to investigate novel diagnostic and therapeutic targets for PTC and explore more detailed molecular mechanisms in the carcinogenesis and progression of PTC. Based on the TCGA and GEO databases, FAM111B is downregulated in PTC tissues and predicts better prognosis in PTC patients. FAM111B suppresses the growth, migration, invasion and glycolysis of PTC both *in vitro* and *in vivo*. Furthermore, estrogen inhibits FAM111B expression by DNMT3B methylation via enhancing the recruitment of DNMT3B to FAM111B promoter. DNMT3B-mediated FAM111B methylation accelerates the growth, migration, invasion and glycolysis of PTC cells. In clinical TC patient specimens, the expression of FAM111B is inversely correlated with the expressions of DNMT3B and the glycolytic gene PGK1. Besides, the expression of FAM111B is inversely correlated while DNMT3B is positively correlated with glucose uptake in PTC patients. Our work established E2/DNMT3B/FAM111B as a crucial axis in regulating the growth and progression of PTC. Suppression of DNMT3B or promotion of FAM111B will be potential promising strategies in the estrogen induced PTC.

## Introduction

Over the past decades, the incidence of TC rapidly increased all over the world 1. The most common histological type of TC is papillary thyroid cancer (PTC), and the other minor histological types include follicular, anaplastic, and medullary TC 2. Moreover, PTC was reported to be the only TC subtype that increased in all countries and has become one of the fastest increasing malignancies. Although the 5-year survival rate for PTC patients is larger than 95%, PTC can be turned into a more aggressive phenotype that metastasizes to lymph nodes or distant tissues and dedifferentiated into more deadly TC 3. Due to the rapidly increasing incidence rate and recurrence of PTC, it is urgent and crucial to discover novel therapeutic targets and agents and to explore more specific molecular mechanisms over thyroid carcinogenesis.

Most malignant thyroid neoplasms are more common in females than in males, especially in adolescence. The incidence of TC is high in females and decreases after menopause 4. Estrogen is a strong development factor for malignant thyroid cells that may account for the gender distinction in the prevalence of TC 5-7. The growth-promoting effect is exerted by estrogen through a non-genomic pathway and a classical genomic pathway. In addition, angiogenesis and metastasis, which are critical for the outcome of TC, are also regulated by estrogen. Previous researches have reported the association between estrogen and PTC, however, the detailed regulatory mechanism of how estrogen induces PTC tumorigenesis and development remains poorly characterized.

FAM111B, the gene of which is positioned on human chromosome 11q12.1, encodes a protein characterized by a trypsin-like serine/cysteine peptidase domain localized at the C-terminus of the protein. The mutation of c.A1873C in FAM111B, resulting in the mutation p.Thr625Pro, links to hereditary fibrosing poikiloderma (HFP) 8. In recent years, aberrant expression of FAM111B has been found to be associated with the occurrence, development and prognosis of various types of neoplasm, such as pancreatic cancer, cervical cancer, prostate cancer, etc. 9. However, the pathological role and biological functions of FAM111B in PTC remain obscure.

In this study, FAM111B was screened out as a novel negatively-correlated gene, which regulated PTC growth and progression and predicted a good clinical outcome of PTC. FAM111B suppresses PTC cell glycolysis, proliferation as well as progression both *in vitro* and *in vivo*. Estrogen inhibits FAM111B expression by enhancing methylation, which was executed by DNA methyltransferase family member DNMT3B, and by recruiting DNMT3B to FAM111B promoter. Moreover, the DNMT inhibitor SGI-1027 dampens PTC cell glycolysis, growth and metastasis via suppression of FAM111B methylation. Thus, our work establishes DNMT3B/FAM111B axis as a crucial and novel axis to modulate PTC growth and progression.

## Materials and Methods

### Cell culture, plasmids, RNA oligonucleotides, lentivirus and Reagents

Human thyroid cancer cell lines (TPC-1 (RRID: CVCL_6298), B-CPAP (RRID: CVCL_0153)) were bought from the American Type Culture Collection (Manassas, VA, USA) and identified based on the corresponding STR profiles (Fig. S1-2). All PTC cell lines were cultivated in RPMI-1640 medium with 10% fetal bovine serum (FBS), with addition of 100 μg/ml streptomycin and 100 U/ml penicillin and placed in a 5% CO_2_ incubator at 37 °C. The eukaryotic expression vector of FAM111B was generated by inserting PCR-amplified fragments into pcDNA3.0. The target siRNA and/or shRNA sequences for FAM111B (JTS Scientific) are listed in Table S1. The sequence for FAM-R (re-expression) was mutated at the siRNA regions (Table S2). After introducing the FAM111B shRNA fragment into the pSIH-H1-Puro vector (System Biosciences), the lentiviral vector expressing FAM111B shRNA was constructed. HEK293T cells were cotransfected with recombinant lentivirus vector and pPACK Packaging Plasmid Mix (System Biosciences) using VigoFect (Vigorous Biotechnology). Following the manufacturer's instructions, the lentivirus was harvested to infect target cells. The stable cells were selected by puromycin. Lipofectamine™ 2000 (Cat# 12566014, ThermoFisher Scientific) and Lipofectamine RNAiMAX (Cat# 13778150, ThermoFisher Scientific) were utilized to conduct plasmid or siRNAs transfection, respectively, as directed by instructions from the manufacturer. Anti-PGK1 (Rabbit Polyclonal; Cat# 17811-1-AP, 1: 100) was purchased from Proteintech. Anti-FAM111B (Rabbit Polyclonal; Cat# YT1669, 1: 100) was purchased from Immunoway. Anti-DNMT3B (Rabbit Monoclonal; Cat# 57868S, 1: 100) was obtained from Cell Signaling Technology. Anti-Ki67 (Rabbit polyclonal; Cat# GB111499, 1: 500) was obtained from Servicebio. The universal secondary antibody (Cat# M21008, 1: 2000) was purchased from Abmart. A 50 mmol/l stock solution of SGI-1027 (Cat# S7276, Selleck Chemicals) and 10 mmol/l stock solution of E2 (Cat# S1709, Selleck Chemicals) were prepared in dimethylsulfoxide (DMSO). The stock solution was preserved at ‑20˚C for long-term storage.

### Analysis of TCGA and GEO dataset

RNA-Sequencing data from TCGA-THCA (Thyroid Cancer patients) was downloaded from the Cancer Genome Atlas (https://portal.gdc.cancer.gov/repository). The data of GSE151179 can be downloaded from the GEO database (https://www.ncbi.nlm.nih.gov/geo/query/acc.cgi?acc = GSE151179). In total, 509 PTC samples (all samples are BRAF mutated) with elaborated expression data were selected from the TCGA database based on parameters that were mentioned above. Elaborated demographics of the patients were depicted by the TCGA consortium.

### Isolation of RNA and analysis of reverse transcription quantitative-PCR (qRT-PCR)

Total RNA was isolated utilizing TRizol reagent (ThermoFisher Scientific) according to the manufacture's protocol. qRT-PCR assay was carried out using the SYBR Green on the system of CFX^96^ (BioRad Laboratories Incorporated). The volume of the total reaction system is 20 μl, which includes 9.0 μl diluted template, 10 μl 2 × SYBR Green I, 0.5 μl sense and 0.5 μl antisense primer. The comparative cycle threshold method (2^‑ΔΔCt^) was used to calculate the relative expression of the target genes, which were normalized to their corresponding internal controls (β-actin). The primers of qRT-PCR used in this experiment were displayed in Table S1.

### Glucose uptake, lactate production and ATP measurement

The measurements of glucose uptake, lactate production and ATP level were performed using the glucose uptake colorimetric assay kit (Cat# K676, Biovision), lactate production assay kit II (Cat# K627, Biovision, USA), and ATP colorimetric assay kit (Cat# K354, Biovision, USA), respectively.

Regarding glucose uptake, cells were plated, regularly cultured and transfected with the indicated constructs. After 48 h, 96-well plates were seeded with 10,000 cell/well and then incubated for 10 h. Cells were rinsed 3 times with PBS, and starved for glucose by incubation in advance of 100 μl Krebs-Ringer-phosphate-HEPES (KRPH) buffer with 2% BSA for 40 min. 10 μl of 10 mM 2-deoxy-D-glucose (2-DG) was added and mixed for 30 min. To remove the exogenous 2-DG, cells were rinsed with PBS for 3 times. Cells were frozen once after being lysed in 90 μl extraction buffer and heated at 85°C for 40 min. Cell lysates were cooled on ice for 5 min, and then, 10 μl neutralization buffer was added. Subsequently, the sample was taken and diluted 10 times by assay buffer. By centrifugating at 12,000 rpm for 5 min, the supernatant was analyzed at 412 nm by microplate reader (Multiscan FC, Thermofisher, USA) to measure the cell glucose uptake. The final glucose uptake results were normalized by the number of cells.

To measure the lactate production, cells were transfected and collected as the above-mentioned glucose uptake analysis. Cells were cultured in RPMI-1640 with 10% FBS for 10 h in the 12-well plates. In order to measure the freshly secreted lactic acid, cells were replaced and incubated with FBS-free RPMI-1640 medium. Supernatants were harvested to measure the lactate production after incubation for 1 h. A total 50 µl of reaction mixture including the following components was prepared: 2 µl Lactate Substrate Mix, 2 µl Lactate Enzyme Mix and 46 µl Lactate Assay Buffer. The total mixture was added to each well and mixed with the lactate standard or test sample. The reaction mixture was added to each well and the 12-well plate incubated for 30 min at room temperature. After incubation, the resultant solutions were measured at 450 nm, and the results was normalized by cell number.

For ATP content measurement, cells were transfected according to the glucose uptake analysis. Cells were collected and isolated in 100 μl Buffer for ATP assay and centrifuged for 5 min at 12, 000 rpm. The harvested supernatant was utilized to detect the ATP level. The mixtures in each well were prepared as follows: 44 µl ATP Assay Buffer, 2 µl ATP Converter, 2 µl ATP Probe and 2 µl Developer. Reaction mixture at 50 µl in each well was incubated with addition of standard ATP sample and test samples. The mixture of reaction, avoided from light, was cultured for 30 min at room temperature. Microplate reader was utilized to estimate lactate levels at 450 nm and normalized by cell number.

### Analysis of Extracellular Acidification Rate (ECAR) and Oxygen Consumption Rate (OCR)

The Seahorse XFe^96^ Extracellular Flux Analyzer (Agilent Technologies, USA) was employed to detect ECAR and OCR with Glycolysis Stress Test Kit and Cell Mito Stress Test Kit (Agilent Technologies, USA), respectively. Both analyses were conducted following the instructions of manufacturer. In brief, the transfected cells were collected and counted for the cell number. ECAR and OCR were measured by seeding 10, 000 cells/well into the Seahorse XF^96^ cell culture microtiter plate. Glucose, oligomycin and 2-DG were subsequently added into the indicated wells at particular time points for ECAR measurement. For OCR assay, oligomycin, FCCP (the reversible inhibitor of oxidative phosphorylation FCCP), and Rote/AA (the mitochondrial complexⅠinhibitor rotenone plus the mitochondrial complex Ⅲ inhibitor antimycin A) were added into indicated wells at particular time points. The data was measured by Seahorse XFe^96^ Wave software. The data of ECAR is recorded in MPH/min while OCR is recorded in pmols/min.

### Analysis of cell growth and colony formation

The Cell Counting Kit-8 (CCK-8) was used to analyze cell proliferation. Each cell well was treated with 10 μl CCK-8 solutions at various time points (0 h, 24 h, 48 h, 72 h and 96 h). Finally, the absorbance was estimated at 450 nm wavelength after 1 h incubation. Cells were seeded in the 3.5 cm plates (3, 000 cells per well) and cultivated in a 5% CO_2_ incubator at 37 °C to perform the colony formation assay. Two weeks later, the colonies were stained with crystal violet for 30 min after being fixed with 4% paraformaldehyde. The colonies were photographed and counted after washing three times with PBS.

### Analysis of cell migration and invasion

Wound healing assay was carried out to evaluate the cell migration. Cells were scratched with a 200 µl pipette tip when grown to 90% confluency on the 6-well plates, and suspended cells were washed off with PBS. The cells were cultured for twenty-four hours to close the wound in the incubator at 37℃. The rates of wound healing were evaluated and generated by comparison with the width at zero hour.

According to the manufacturer's protocol, the cell invasive ability was determined using the Matrigel Invasion Chambers (BD Biosciences). Cells were plated into the upper well in a 5% CO_2_ incubator at 37 °C. 24 h later, the chambers were stained with crystal violet for about 30 min after being fixed with 4% paraformaldehyde. The invasive cell number was analyzed under a magnification microscope in the bottom of the chamber and photographed 10.

### *In vivo* analysis of tumor growth and metastasis

All the animal experiments conducted in this study were approved and guided by the Ethics Committee of Beijing Institute of Biotechnology. Experimental mice were raised in a specific pathogen-free house. 1 × 10^7^ TPC-1 cells harboring various constructs were subcutaneously injected into the right flanks of nude mice for *in vivo* tumor evaluation. The tumor volumes were calculated every 5 days using caliper (tumor volumes = 1/2 × length × width^2^), and the tumor growth curve was plotted. All the mice were killed on day 45, and all the tumors were excised and then stored at -80°C.

The nude mice were injected with 1 × 10^6^ TPC-1 cells harboring the corresponding constructs via the lateral tail vein. To examine the metastasis of lung, all mice were sacrificed on day 50. Then, the CT scan at the location of the lung in each group was conducted and analyzed for representative metastatic foci of the lungs. The characteristic lung metastases were selected and dissected, under formaldehyde fixation and H & E staining. The number of lung metastasis nodules was determined by lung CT scan.

### Immunohistochemistry (IHC) staining

Forty-one human TC samples (all patients were BRAF mutated and female) were acquired from Beijing Haidian Hospital and Peking University First Hospital with the approval from both hospitals. IHC was performed on the paraffin-embedded and formalin-fixed clinical tissue samples. Tissue pieces were initially deparaffinized, rehydrated and incubated with 3% H_2_O_2_ for 15 min to restrain the activity of endogenous peroxidase. After the recovery of the epitope in 10 mM citrate buffer (pH 6.0) by microwaving for 30 min, the primary antibodies solution was added and incubated at 4°C overnight [anti-PGK1 (1: 100), anti-FAM111B (1: 50) and anti-DNMT3B (1: 50)]. And then, 100 µl of biotin-labeled goat anti-mouse/rabbit IgG polymer (Cat#SP-9000, ZSGB-BIO, China) was added for the second step incubation, and finally 3, 3'-diaminobenzidine tetrachloride was used to generate a signal.

Expression of the sections was separately evaluated by a different pathologist who didn't know the information of the patients from the clinic. Scores of FAM111B, DNMT3B and PGK1 were obtained as follows: multiplying the percentage of stained cells (0 ~ 100%) by staining intensity (low, 1+; medium, 2+; strong, 3+). Therefore, the range of score is 0 ~ 3. The cut-off values of the IHC score were estimated according to receiver operating characteristic (ROC) curve analysis. For further correlation analysis, the samples scored < 0.5 and scored ≥ 0.5 were considered too low and too high FAM111B, while the samples scored < 0.75 and scored ≥ 0.75 were considered too low and too high DNMT3B and PGK1.

### Animal PET studies

PET imaging of mice was acquired by utilizing an animal PET scanner (Philips Incorporated). Prior to the PET scan, mice were fasted overnight and were allowed to drink freely. By inhaling of 5% isoflurane/oxygen mixture, mice were anesthetized, then the mice were placed on an inclined pre-warmed mouse bed and injected 3.7 MBq (100 μCi) of ^18^F-FDG fluorodeoxyglucose through intravenous tail-vein injection. 60 min after injection, 5 min emission scans were conducted to obtain attenuation correction data in the prone position, and 10 min delayed scan was performed within 2 h.

### Statistical Analysis

SPSS 23.0 and GraphPad Prism 8.0 were used for statistical analyses used in this study. The statistical significance of cell proliferation, migration and invasion were determined using the two-tailed Student's t test. Spearman correlation analysis was carried out using GraphPad Prism 8.0 software to access the relationship between the variables. P-values less than 0.05 were regarded as statistically significant. All values were shown as mean ± SD of triplicate measurements and have been repeated 3 times with similar results.

## Results

### FAM111B is selected as one of the most significantly expressed genes in PTC tissues and predicts better prognosis in PTC patients

**Figure 1:**
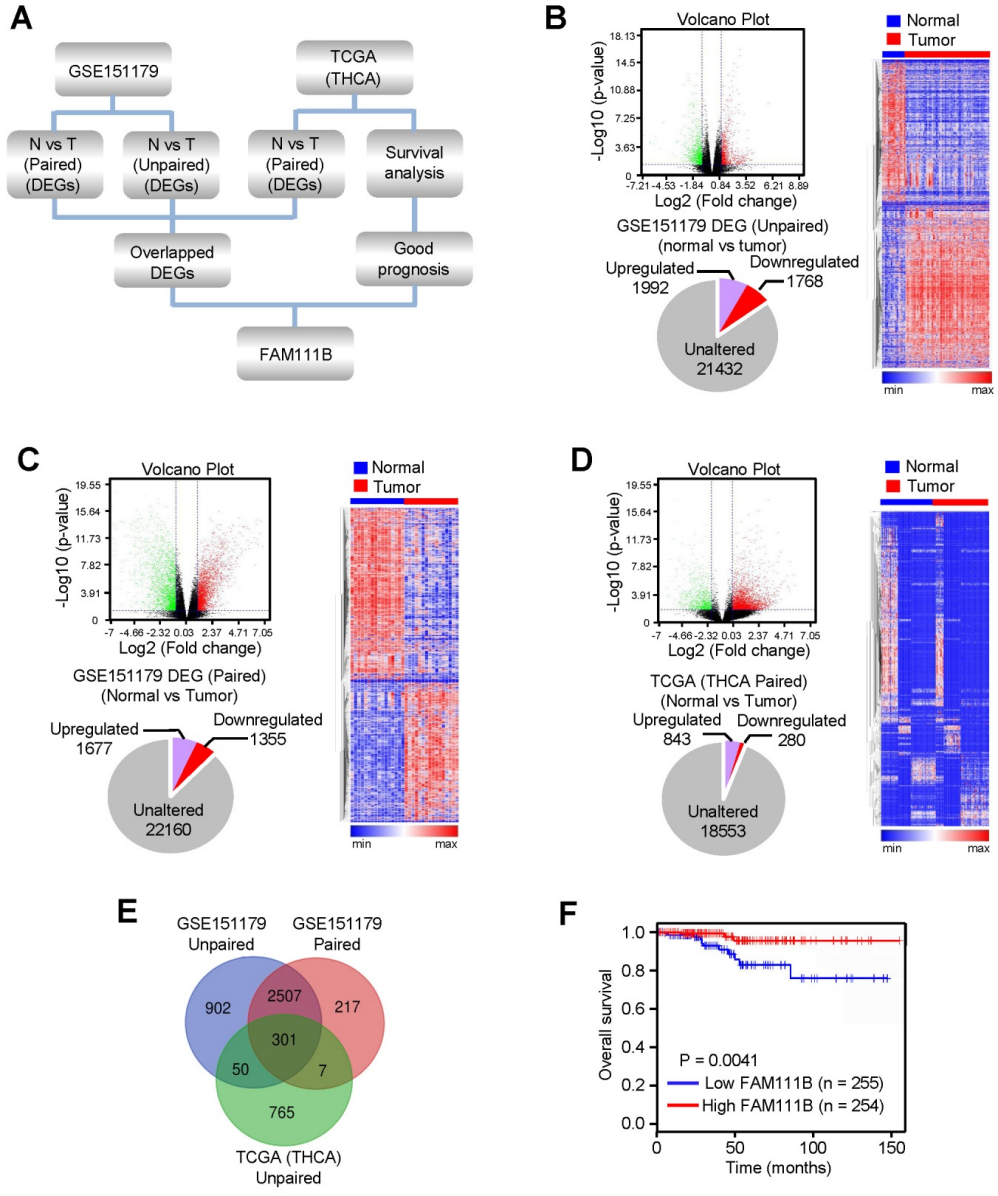
** FAM111B is selected as one of the most significantly expressed genes in PTC tissues and predicts better prognosis in PTC patients.** (A) Flow chart displaying the screening strategy for crucial genes in PTC. (B) DEGs between normal and tumor tissues from GSE151179 dataset shown in the volcano plot. Downregulated and upregulated genes were displayed in green and red, respectively. Values were demonstrated as log2 of tag counts. A total of 25192 genes, of which 1992 were upregulated and 1768 were down-regulated genes displayed in the pie chart. All statistically significant DEGs were demonstrated in the hierarchical cluster analysis diagram. (C) Paired GSE151179 and THCA DEGs expression analyzed as in (B). (D) Paired THCA expression analyzed as in (B). (E) Venn analysis of the DEGs according to the strategy. The DEGs in each screening strategy were represented as three circles, and the intersection of the results was represented as the middle part. (F) The difference in OS between patients with high and low expression of FAM111B in PTC from TCGA database by Kaplan Meier analysis. Censored samples were represented by marks on graph lines.

In order to explore novel genes that have the potential to suppress the growth and progression of PTC, RNA-Seq data of PTC from the GEO database (GSE151179) and TCGA database were analyzed following the strategy illustrated in Figure 1A. In brief, the differentially expressed genes (DEGs) were screened between cancerous tissues and their normal counterparts in GEO and TCGA databases. 3760 (1992 upregulated and 1768 downregulated) and 3032 (1677 upregulated and 1355 downregulated) DEGs were identified from GSE151179, based on unpaired or paired normal and tumor tissues, respectively, and 1123 (843 upregulated and 280 downregulated) DEGs were identified between paired normal and tumor tissues according to THCA data of TCGA database (Fig. 1B-D). Eventually, we obtained 301 differentially crossed genes, 227 of which were upregulated and 74 were downregulated (Fig. 1E). Among the 74 downregulated genes, we found the high expression of FAM111B gene exhibited the best overall survival (OS) in PTC according to the survival analysis in the database of GEPIA, which was therefore selected for further study (Fig. 1F). In conclusion, the above-listed data suggests that FAM111B may be a novel negatively-regulated gene involved in the carcinogenesis of PTC.

### FAM111B suppresses PTC growth, migration and invasion* in vitro* and *in vivo*

**Figure 2:**
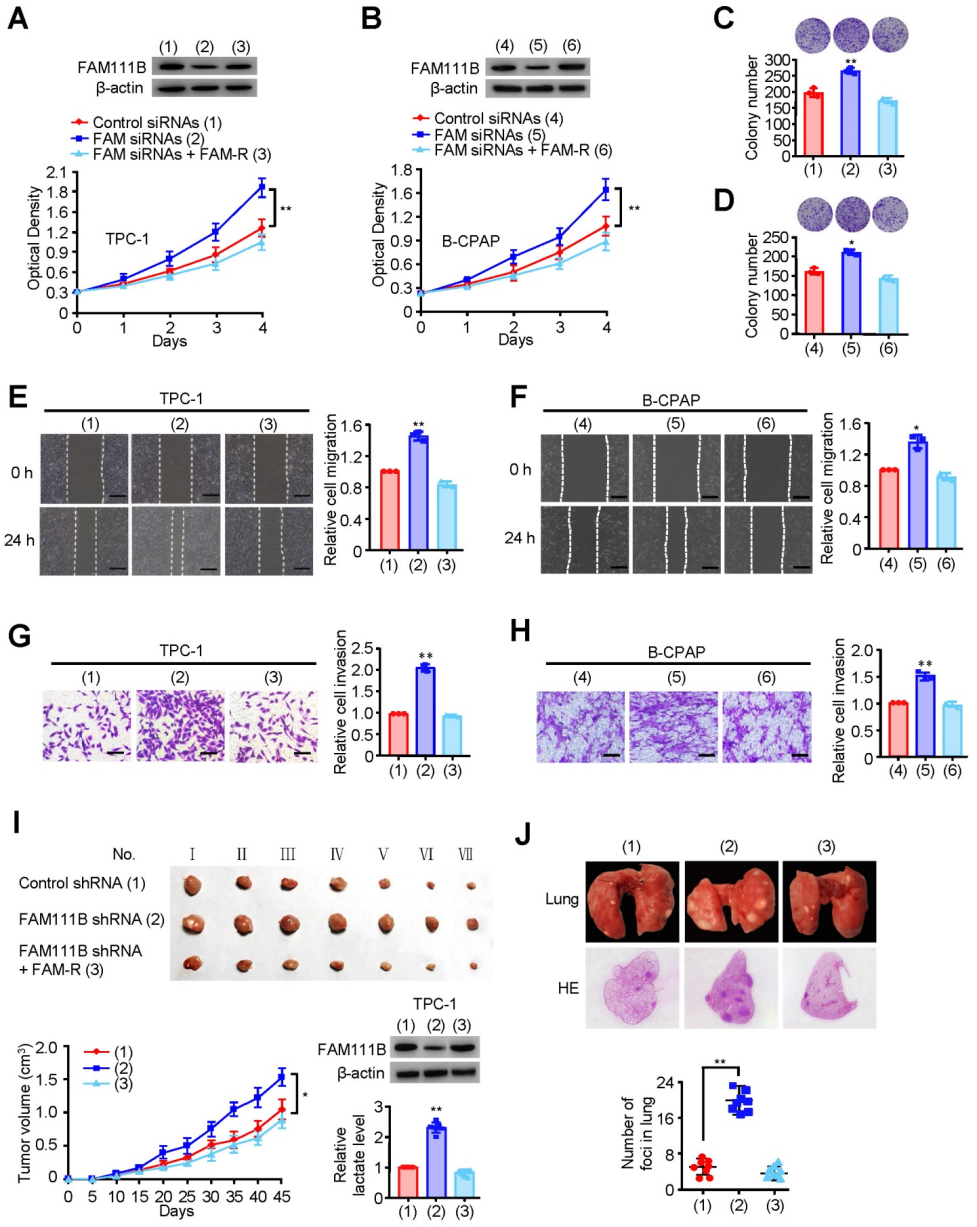
** FAM111B suppresses PTC growth, migration and invasion* in vitro* and *in vivo*.** (A, B) TPC-1 cells and B-CPAP cells were transfected with control siRNA, FAM111B siRNAs or FAM111B siRNAs plus FAM111B. Western blot analysis was used to detect the expression level of FAM111B protein. The cell proliferation was determined by the CCK-8 assay at 450 nm (OD450) with a microplate reader. (C, D) Representative images indicates the colonies in plates (upper panels). The histogram indicates the colony number. (E, F) Wound healing assay of TPC-1 cells and B-CPAP cells transfected as in (A, B). Representative histograms indicate relative cell migration. (G, H) Transwell assays of TPC-1 cells and B-CPAP cells transfected as in (A, B). Representative histograms indicate relative cell invasion. All values displayed are mean ± SD and have been duplicated 3 times with similar results (A-H). *p < 0.05 and **p < 0.01 versus corresponding Control siRNA. (I) TPC-1 cells stably infected with lentivirus carrying the indicated constructs were injected into nude mice (n = 7 per group) as indicated. After 45 days, mice were sacrificed to harvest tumors. At the indicated times, tumors were measured (mean ± SD, n = 7), and the growth curve was plotted. The representative immunoblot indicates the FAM111B expression and the histogram displays the production of lactate. (J) TPC-1 cells expressing the indicated constructs were injected through the tail vein to build a PTC cell metastasis model in nude mice (n = 8 per group). Lung CT scan of groups were performed and representative metastatic foci of lungs were subjected to anatomical and histological analyses. *p < 0.05 and **p < 0.01 vs. control shRNA group. Scale bar, 50 μm.

As database analysis suggests that the expression of FAM111B exhibits clinical importance in PTC patients, we investigated whether FAM111B mediated the growth, migration, and invasion in cultured PTC cells. TPC-1 and B-CPAP cells transfected with FAM111B siRNAs grew more rapidly than the corresponding control cells. FAM111B re-expression could reverse this effect in these two FAM111B siRNAs transfected PTC cells (Fig. 2A, B). Colony formation displayed similar trends to the above-mentioned growth curve (Fig. 2C, D). These data suggest that FAM111B suppresses PTC cell growth. Subsequently, wound-healing and transwell assays were conducted to determine the migratory and invasive abilities of FAM111B in both cells (Fig. 2E-H). The results showed that FAM111B knockdown enhanced the migration and invasion of PTC cells while FAM111B re-expression could restore the effect of FAM111B knockdown.

To study the phenotype of FAM111B *in vivo*, TPC-1 cells carrying the control shRNA, FAM111B shRNA, or FAM111B shRNA plus FAM111B were subcutaneously injected into the right side of female nude mice. The result showed that the tumors with FAM111B depletion cells grew faster than those of the control cells. Moreover, the knockdown effect of FAM111B was reversed by FAM111B re-expression to control the tumor xenograft growth (Fig. 2I). Next, the effect of FAM111B on PTC cell metastasis was examined. The number of nodules that diffused within the pulmonary region was significantly elevated in the FAM111B knockdown group in comparison with that in the control group. Importantly, FAM111B markedly reversed the effect of FAM111B knockdown to suppress lung metastasis (Fig. 2J). The metastasis foci were confirmed by histological analysis of the lungs. Overall, these experimental results suggest that FAM111B may play a suppressive function in the growth, migration and invasion of TPC-1 and B-CPAP cells both* in vitro* and* in vivo*.

### FAM111B inhibits glycolysis in PTC cells* in vitro* and* in vivo*

**Figure 3:**
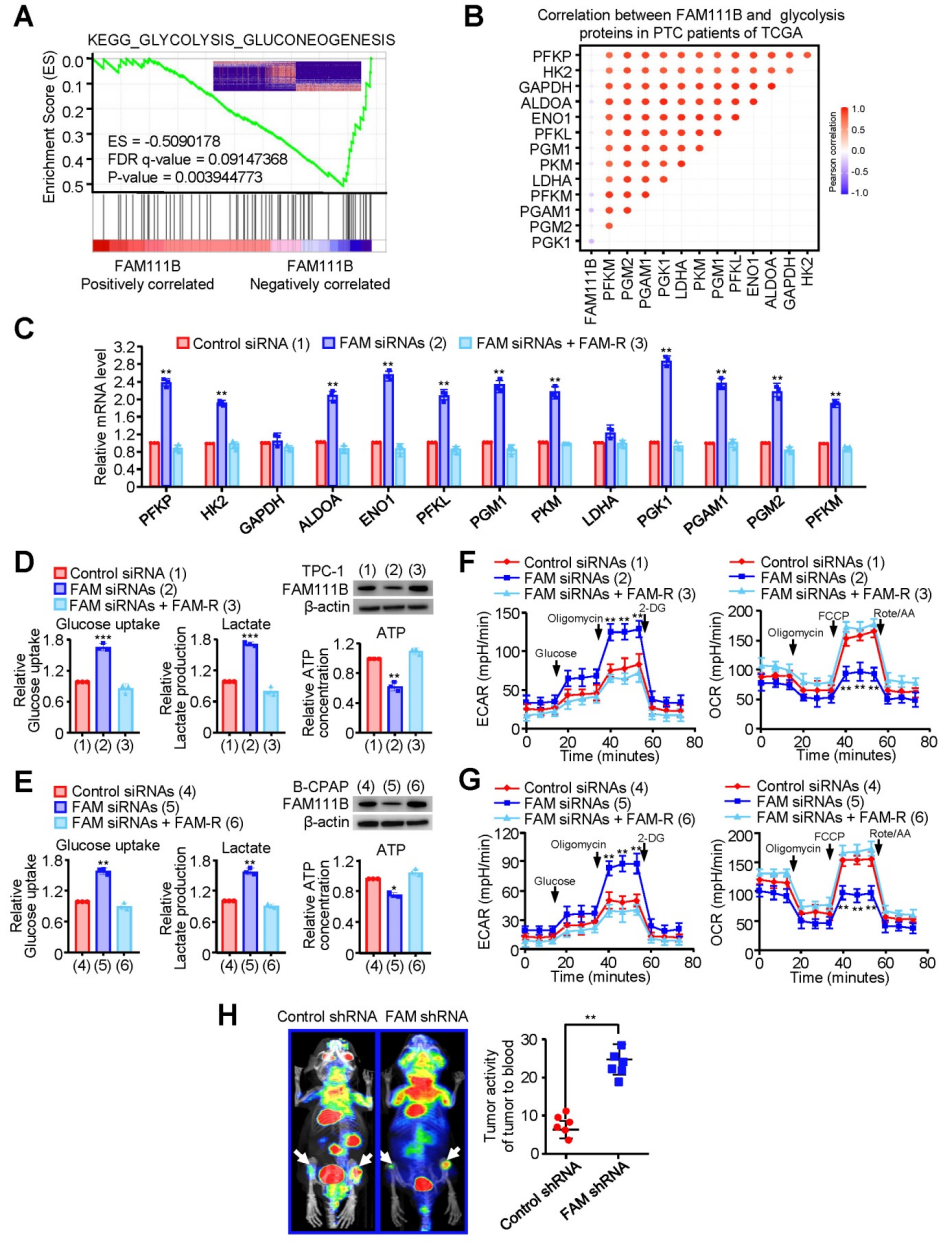
** FAM111B inhibits glycolysis in PTC cells *in vitro* and *in vivo*.** (A) GSEA plot showing that FAM111B expression is positively correlated with Warburg effect signaling in the TCGA THCA dataset. (B) Correlation between FAM111B and glycolysis genes in PTC patients of TCGA was valued. (C) The mRNA expression of glycolytic genes (PFKP, HK2, GAPDH, ALDOA, ENO1, PFKL, PGM1, PKM, LDHA, PGK1, PGAM1, PGM2, PFKM) in TPC-1 cells with control siRNA or FAM111B siRNAs or FAM111B siRNAs plus FAM-R. (D, E) TPC-1 and B-CPAP cells were transfected with control siRNA or FAM111B siRNAs or FAM111B siRNAs plus FAM-R respectively. Glucose uptake, lactate production and ATP production were measured. Typical immunoblot indicates the expression of FAM111B. (F, G) TPC-1 cells (F) and B-CPAP cells (G) were transfected as in (D), and the extracellular acidification rate (ECAR) and oxygen consumption rate (OCR) were then evaluated. The arrows indicate the time of adding glucose, oligomycin, 2-DG, oligomycin, FCCP and rotenone and antimycin A (Rot/AA). All values shown are mean ± S.D. of triplicate measurements and have been repeated 3 times with similar results (d-g). *p < 0.05, **p < 0.01, ***p < 0.001 vs. Control siRNA group. (H)^18^F-FDG PET scan performed before sacrifice of mice in each group (n = 6 per group). The activity of tumor to blood was measured in each group. **p < 0.01 versus Control shRNA group.

To deeply investigate the molecular mechanism by which FAM111B regulates the growth and progression of PTC cells, gene set enrichment analysis (GSEA) was performed on the RNA-seq data of thyroid cancer in the TCGA database. Interestingly, lower FAM111B expression was positively correlated with glycolysis-gluconeogenesis, and higher FAM111B expression was positively correlated with cell cycle arrest and negative regulation of cell cycle process (Fig. 3A & Supplementary Fig. 3A, B). In addition, a significant negative correlation was observed between the expression of FAM111B and the expression of a set of glycolytic-related gene expression, which was further verified by qRT-PCR assay (Fig. 3B, C).

To verify the effect of FAM111B on glycolysis in PTC cells, we measured the glucose uptake, lactate and ATP production. In comparison with the cells transfected with control siRNA, TPC-1 and B-CPAP cells transfected with FAM111B siRNAs exhibited increased glucose uptake, increased lactate production and decreased ATP production. As expected, re-expression of FAM111B reversed these effects (Fig. 3D, E). Extracellular acidification rate (ECAR) and oxygen consumption rate (OCR) were used to detect the ability of extracellular acid production and oxidative phosphorylation of cells, to indirectly and directly reflect the changes of glycolysis 10.

The results showed that TPC-1 and B-CPAP cells transfected with FAM111B siRNAs increased the ECAR and reduced the OCR in comparison with the cells that transfected with control siRNA and restoration of FAM111B could reverse these effects (Fig. 3F, G). To further verify the molecular mechanism of FAM111B controlling the growth and migration of PTC cells, we utilized the glycolytic inhibitor, 2-deoxy-D-glucose (2-DG). The results showed that 2-DG greatly inhibited the ability of FAM111B knockdown to accelerate proliferation and migration in PTC cells, suggesting that FAM111B controls the growth and migration of PTC cells largely dependent on glycolysis (Fig. S3C, D). To examine the effects of FAM111B on glycolysis *in vivo*, PET (positron emission tomography) scans were employed to evaluate the glucose uptake in tumor xenografts of nude mice. Increased glucose uptake was observed in the tumors with FAM111B knockdown compared with that of the control group, indicating FAM111B suppresses PTC cell glycolysis *in vivo* (Fig. 3H)*.*

### Estrogen inhibits FAM111B expression by DNMT3B methylation via enhancing the recruitment of DNMT3B to FAM111B promoter

**Figure 4:**
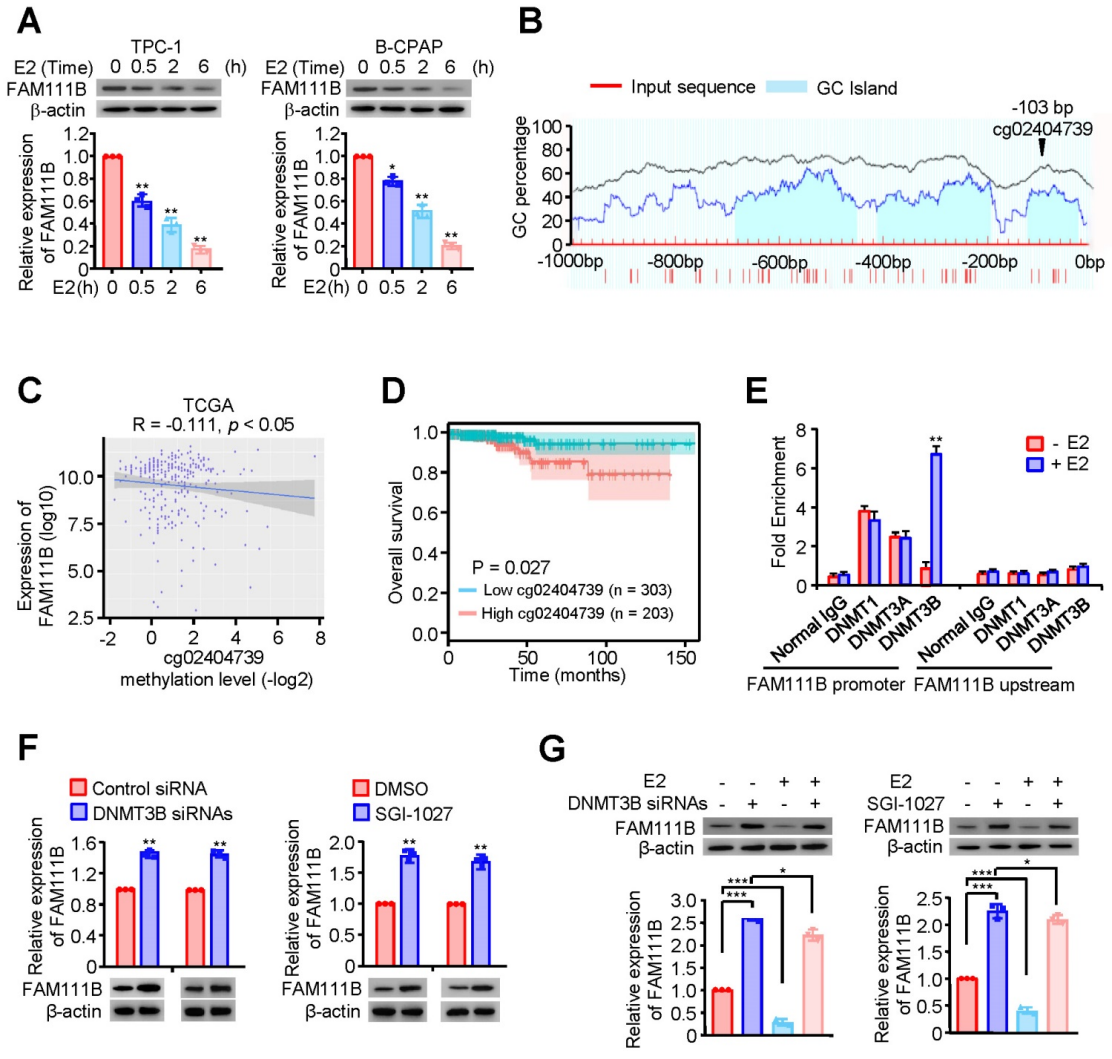
** Estrogen inhibits FAM111B expression by DNMT3B methylation via enhancing the recruitment of DNMT3B to FAM111B promoter.** (A) Western blot analysis of TPC-1 and B-CPAP cells with E2 at different times (0 h, 0.5 h, 2 h, 6 h) were performed. The representative immunoblot shows the expression of FAM111B. Lower panels show the relative levels of FAM111B protein density. (B) Schematic of the CpG islands in the FAM111B promoter. Input sequence: red region; CpG islands: blue region; cg02404739: the CG sites in the FAM111B CpG islands, identified using the TCGA dataset. (C) The relationship between cg02404739 methylation levels and FAM111B expression was assessed using Spearman's rank correlation analysis. Symbols represent individual samples. (D) Kaplan-Meier analysis of the correlation between the cg02404739 levels and overall survival of PTC patients with high (n = 203) and low (n = 303) cg02404739 expression in the TCGA cohort. (E) ChIP analysis of DNA methylases occupancy on the FAM111B promoter or upstream of the promoter in TPC-1 cells treated with E2 or not. **p < 0.01 versus E2 absent group. (F) Immunoblot analysis of FAM111B protein levels in the TPC-1 cells transfected with DNMT3B siRNAs or treated with SGI-1027. Upper panels show the relative levels of FAM111B protein density. (G) qRT-PCR analysis of FAM111B mRNA and immunoblot analysis of FAM111B protein in the TPC-1 cells transfected with DNMT3B siRNAs or treated with or without SGI-1027 exposed to E2 or not. Lower panels show the relative levels of FAM111B protein density. The triplicate measurement results were repeated 3 times and the results were similar. *p < 0.05, **p < 0.01, ***p < 0.001 versus the corresponding control group.

Estrogen (E2) is a potent growth factor acknowledged as a promoter of PTC cell growth and progression, yet its molecular mechanisms are poorly characterized 7. Initially, we investigated whether E2 regulated glycolysis in PTC cells. B-CPAP cells and TPC-1 were dealt with DMSO or E2, then glucose uptake, lactate and ATP production as well as ECAR were measured. The results indicated that E2 upregulated glycolysis in PTC cells (Supplementary Fig. 4A-D). Therefore, we considered whether estrogen affects FAM111B expression. Interestingly, the expression of FAM111B decreased with the addition of E2 at different time in both TPC-1 and B-CPAP cells (Fig. 4A). Studies have reported that E2 is related to the methylation level of the whole genome. Among the three possible methylation sites, only cg02404739 is located at the transcription start site (Fig. 4B). Next, we analyzed the methylation sites of FAM111B promoter in TC based on the TCGA database and found 10 possible methylation modification sites in the upstream of FAM111B, of which 3 sites had a *P* value that was smaller than 0.01 in univariate analysis and were negatively correlated with FAM111B expression (Fig. 4C and Table 1). Survival analysis further confirmed that the methylation of the cg02404739 site predicted poorer prognosis in TC patients (Fig. 4D). Chromatin immunoprecipitation (ChIP) assay demonstrated that only DNMT3B recruits to the promoter region of FAM111B, indicating that DNMT3B might be the DNA methylase responsible for FAM111B promoter methylation (Fig. 4E). To verify whether DNMT3B actually regulates FAM111B expression, we employed DNMT3B siRNAs or SGI-1027, the inhibitor of DNMT3, into TPC-1 cells and estimated the expression level of FAM111B. As expected, FAM111B expression was significantly upregulated under either DNMT3B siRNAs or SGI-1027 treatment (Fig. 4F). In order to clarify the molecular mechanism by which E2 downregulates FAM111B expression, we investigated the effect of E2 on FAM111B expression under DNMT3B siRNAs or SGI-1027 treatment. The results showed that the downregulation of the FAM111B expression by E2 was almost eradicated when DNMT3B was inhibited by DNMT3B siRNAs or SGI-1027 (Fig. 4G), indicating that E2 downregulates FAM111B expression by DNMT3B methylation via enhancing the recruitment of DNMT3B to FAM111B promoter.

### Methylation of FAM111B by DNMT3B promotes the growth, migration, invasion and glycolysis of PTC

**Figure 5:**
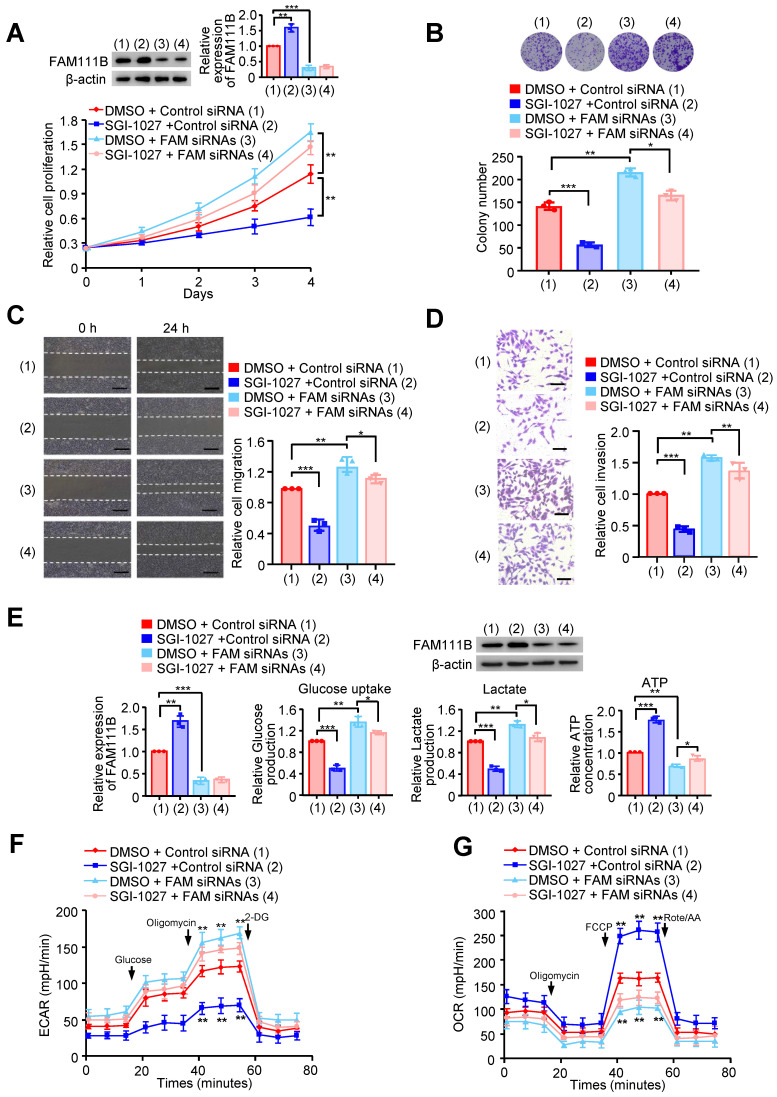
** Methylation of FAM111B by DNMT3B promotes the growth, migration, invasion and glycolysis of PTC.** (A) TPC-1 cells were transfected with Control siRNA or FAM111B siRNAs and treated with SGI-1027 or not. The proliferation of the cells was detected by CCK-8 assay. The representative immunoblot displays FAM111B protein level. Left panels show the relative levels of FAM111B protein density. (B) Colony formation assay of TPC-1 cells transfected and treated as in (A). Representative images show colonies on plates (upper panels). Histograms display colony number (B). (C, D) Wound healing (C) and transwell (D) assays of TPC-1 cells transfected and treated as in (A). Representative histograms show the relative cell migration and invasion. (E) TPC-1 cells were transfected and treated as in (A), and glucose uptake, the production of lactate and ATP were determined. Representative immunoblot reveals the protein expression of FAM111B. Lower panels show the relative levels of FAM111B protein density. (F, G) TPC-1 cells were transfected and treated as in (A), and extracellular acidification rate (ECAR) (F) and oxygen consumption rate (OCR) (G) were then measured. Scale bar, 25 μm. All values shown are mean ± S.D. of triplicate measurements and have been repeated 3 times with similar results (A-G). *p < 0.05, **p < 0.01, ***p < 0.001 vs. DMSO + Control siRNA.

Next, we investigated whether methylation of FAM111B by DNMT3B affects the phenotype of FAM111B in PTC cell growth, migration, invasion as well as glycolysis. FAM111B was knocked down in PTC cells, and these knocked-down cells were treated with SGI-1027. Cell growth and colony formation assays demonstrated that SGI-1027 inhibited PTC cell growth, whereas FAM111B knockdown enhanced PTC cell growth. Importantly, knocking down of FAM111B greatly impaired the ability of SGI-1027 to affect PTC cell proliferation, indicating that the enhancement of PTC cell proliferation by DNMT3B largely depends on the regulation of FAM111B expression (Fig. 5A, B & Supplementary Fig. 5A, B).

We also investigated whether the methylation of FAM111B by DNMT3B affects the phenotype of FAM111B in PTC cell migration, invasion as well as glycolysis. As expected, SGI-1027 decreased the migration and invasion of PTC cells (Fig. 5C, D and Supplementary Fig. 5C, D). More importantly, the regulatory ability of SGI-1027 was greatly attenuated by FAM111B knockdown to suppress PTC cell migration and invasion. Besides, SGI-1027 lowered the glucose uptake and lactate production levels, increased that of the ATP generation and exhibited upregulated OCR and downregulated ECAR levels. Again, the regulatory ability of SGI-1027 on PTC cell migration, invasion and glycolysis was impaired by FAM111B knockdown (Fig. 5E-G & Supplementary Fig. 5E, F). In conclusion, these results suggest that methylation of FAM111B by DNMT3B promotes the growth, migration, invasion, and glycolysis of PTC.

### Methylation of FAM111B promotes the growth and lung metastasis of PTC *in vivo*

**Figure 6:**
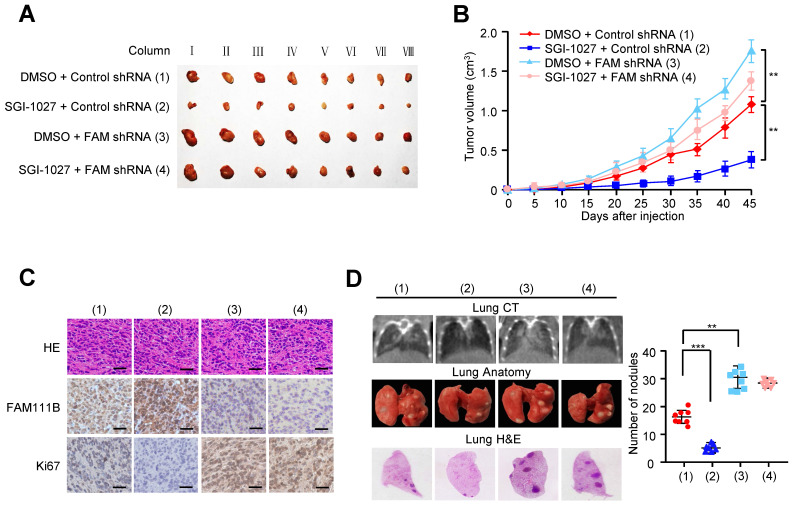
** Methylation of FAM111B promotes the growth and lung metastasis of PTC *in vivo*.** (A, B) TPC-1 cells firmly infected with the lentivirus carrying the indicated constructs were injected subcutaneously into nude mice (n = 8 per group). The tumor volume was evaluated every 5 days and the growth curve was plotted (B). (C) Representative IHC staining of FAM111B, Ki67 and H&E images of tumors resected from (A). Scale bar, 50 µm. (D) TPC-1 cells expressing the constructs were injected through the tail vein to build a PTC cell metastasis model in nude mice (n = 8 per group). Lung CT scan of groups were performed and representative metastatic foci of lungs were subjected to anatomical and histological analyses. **p < 0.01, ***p < 0.001 versus the corresponding control.

In order to verify the *in vivo* phenotype of FAM111B methylation, we examined its effect on PTC growth by injecting TPC cells carrying the indicated constructs into the back of BALB/c nude mice. As expected, FAM111B knockdown remarkably accelerated PTC tumor growth. On the contrary, tumor growth was suppressed when treated with SGI-1027. More importantly, the effect of SGI-1027 treatment on tumor growth was significantly abrogated under FAM111B knockdown (Fig. 6A-C).

We also evaluated the effect of FAM111B methylation on PTC tumor metastasis. The number of nodules that were diffused within the pulmonary region was significantly increased in the FAM111B knockdown group in comparison with that in the control group. On the contrary, SGI-1027 caused the decreased spread of metastatic PTC cells to the lung. Importantly, FAM111B knockdown largely impaired the ability of SGI-1027 to suppress lung metastasis (Fig. 6D). The metastasis foci in the lungs were confirmed by histological analysis. Taken together, these data suggest that methylation of FAM111B promotes the PTC growth and lung metastasis *in vivo*.

### Correlations between DNMT3B, FAM111B and glycolytic gene expression and association of FAM111B and DNMT3B with glucose uptake in thyroid cancer patients

**Figure 7:**
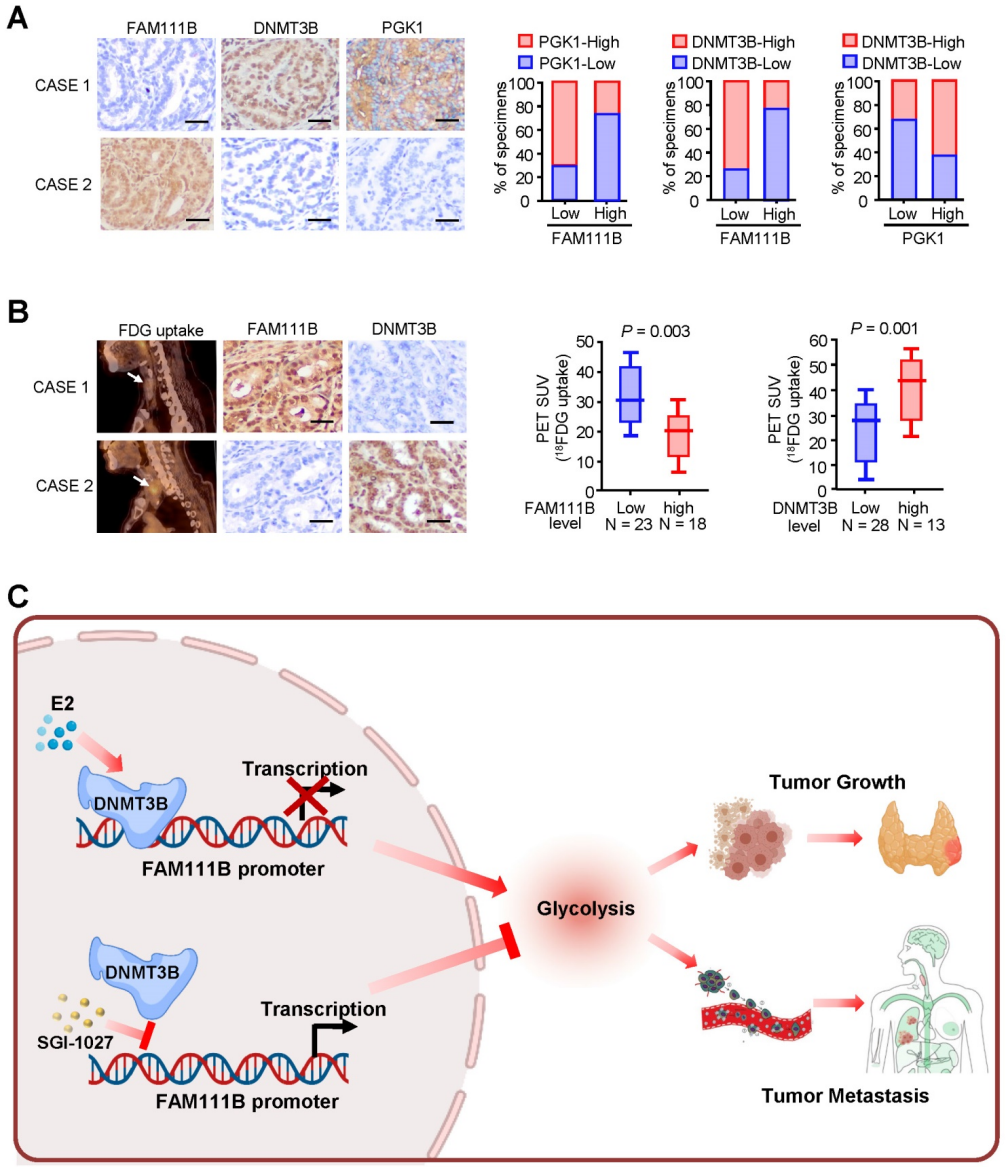
** Correlations between DNMT3B, FAM111B and glycolytic gene expression and association of FAM111B and DNMT3B with glucose uptake in patients with thyroid cancer.** (A) Representative IHC staining for FAM111B, DNMT3B and PGK1 in PTC patients. Scale bar, 50 μm. Right panel showing the percentage of specimens with low or high PGK1 and DNMT3B expressions in the low or high FAM111B expression groups. CASE 1 and CASE 2 refer to two representative samples categorized by low and high FAM111B expression. (B) The correlation of glucose uptake in TC patients with different expressions of FAM111B and DNMT3B using the Mann Whitney *U* test. CASE 1 and CASE 2 refer to two representative samples categorized by low and high FDG uptake. Scale bar, 50 μm. (C) Graphical abstract underlying the role of the DNMT3B/FAM111B axis in regulating PTC growth and lung metastasis.

Immunohistochemical staining (IHC) was used to test the expression of FAM111B, DNMT3B, and PGK1, which is one of the glycolytic genes that was most significantly negatively-regulated by FAM111B, in 41 human PTC patients. In accordance with the findings in cultured cells, FAM111B expression level in PTC tissues was inversely correlated with DNMT3B and PGK1 expression. In addition, DNMT3B expression was positively correlated with PGK1 expression (Fig. 7A). Interestingly, PTC patients who had upregulated glucose uptake scanned by ^18^FDG PET exhibited lower level of FAM111B and higher level of DNMT3B expression (Fig. 7B). The specificity of the FAM111B and DNMT3B antibodies were validated by IHC and immunoblotting with cell lysates (Supplementary Fig. 6). Overall, these data suggest the crucial pathological role of DNMT3B/FAM111B axis in PTC.

## Discussion

Epidemiology and clinical studies have shown that the prevalence of differentiated TC in women was 3-4 fold higher than that in men, and the raised incidence of this cancer that is associated with the onset of puberty in females is well recorded 11, 12. In addition, TC and breast cancer are the two most common malignancies in females. These cancers often successively occur. Women with TC are at an increased risk of subsequent breast cancer; women who have breast cancer are more probable to develop later TC, indicating a usual etiology 13. A key potential pathogenic factor connecting these two diseases is the hormonal signal from the thyroid and estrogen 14. Breast cancer, the most prevalent malignancy in females, was acknowledged to be linked with steroid hormone estrogen. The results of these studies show that estrogen as well as its receptors are involved in regulating the TC growth. Estrogen executes its growth-promoting function through both of a genomic and non-genomic pathway, mediated through a membrane-bound estrogen receptor 7. This receptor is related to the tyrosine kinase signaling pathways, such as MAPK and PI3K 15-17. During the process of PTC carcinogenesis, the chromosomal rearrangement of the tyrosine receptor kinase TRKA, or by a BRAF mutation may induce the activation of these signaling pathways 18. In addition, in females who possess higher levels of estrogen, these pathways are more likely to be stimulated. Estrogen is also related to the modulation of angiogenesis and metastasis that are essential for TC outcome 19-22. However, it is worth noting that the existing population cohort studies have shown that the use of oral contraceptives (OC) is inversely correlated with the risk of PTC in women. This may be related to the presence of progesterone in OC and indicated that the use of OC probably could decrease the risk of PTC. In addition, using only estrogen in hormone replacement therapy increases the risk of TC in females 23-25. Although there have been researches focused on the association between estrogen and PTC, the detailed regulatory mechanisms of how estrogen induces PTC tumorigenesis and development remain still unclear. In this present study, we found that estrogen inhibited the expression of FAM111B by DNMT3B methylation via increasing the recruitment of DNMT3B to the FAM111B promoter, and thus promoted the growth and progression of PTC. This is a novel mechanism of estrogen induction in PTC tumorigenesis and development. Supplement of FAM111B will be a promising therapeutic strategy to prevent estrogen-induced PTC growth and metastasis. However, it is interesting that the TCGA databases analysis results did not exhibit obvious relationship between high expression of FAM111B and the gender ratio. Female PTC patients failed to show a higher FAM111B expression than male patients. It reminds us that estrogen as an upstream regulator may play a more important role in female PTC patients.

Cancer cells are characterized by an altered metabolism, specifically with upregulated glucose uptake and fermentation of glucose to lactate, a phenomenon which is known as the “Warburg Effect” 26-28. This metabolic reprogramming brings a growth advantage for the cancerous cells to enhance survival, proliferation, and progression. Hence, targeting the process of aerobic glycolysis has been acknowledged as an effective approach to control the proliferation and development of a tumor, as well as to enhance the effect of anticancer therapeutics 29. Negative regulators of the glycolytic pathway have attracted great research attention as candidate therapeutic agents of cancer treatment, which motivated researchers to search for novel negative regulators to suppress the glycolysis pathway or lead to a switch from glycolysis to mitochondrial respiration. FAM111B encodes a protein with a trypsin-like cysteine/serine peptidase domain. Most studies of FAM111B were focused on its relationship with hereditary fibrosing poikiloderma (HFP) and relatively few studies concentrated on the effect of FAM111B on human cancers 30, 31. Sun *et al.* reported that FAM111B is related to the p53 signaling pathway and executes its function as an oncogene, implying a helpful therapeutic target in lung adenocarcinoma patients 32. Kawasaki *et al.* reported that FAM111B promotes the proliferation of KRAS-driven lung adenocarcinoma by degrading p16, suggesting a clinicopathological marker for predicting LUAD prognosis 33*.* Nevertheless, the clinicopathological significance of FAM111B and the exact role of FAM111B in PTC are unclear. In our current research, FAM111B was screened out as a novel correlated gene that negatively regulated growth and progression of PTC and predicted better clinical outcome in PTC. FAM111B suppressed PTC cell glycolysis, growth and progression both *in vitro* and *in vivo*. Moreover, estrogen inhibits FAM111B expression by DNMT3B methylation by enhancing the recruitment of DNMT3B to the FAM111B promoter. Thus, our work established FAM111B as a novel negative regulator to suppress PTC glycolysis, growth, and progression.

Researchers have been dedicated to the mechanisms of DNA methylation mediating tumorigenesis for decades. DNA methylation is one of the main topics of epigenetics, which is crucial for accelerating tumor growth, migration, and angiogenesis throughout carcinogenesis 34, 35. Abnormal hypermethylation of CpG islands in tumor suppressor gene's promoter region is one of the ways to inactivate a tumor suppressor gene 36, 37. DNA methyltransferase (DNMT) is related to the transformation or progression of human cancer by mediating hypermethylation of tumor suppressor factors 38. DNMT3B is a member of the DNMT family, which is mainly located in the nucleus, and its expression is regulated by development 39. Its main function is de novo methylation, which can cause the inactivation of tumor suppressor genes 40-42. Current studies have found that DNMT3B is linked to the susceptibility and development of various malignant tumors 9. However, the role of DNMT3B in PTC and methylation during the development of PTC remains poorly characterized. SGI-1027, a DNMT inhibitor, causes selective degradation of DNMT3B in several human cancer cells, such as melanoma, lung carcinoma and breast cancer, but shows little or no cytotoxic effect on some types of cancer cells, such as hepatoma cells 43, 44. Whether SGI-1027 affects PTC cell growth and progression needs to be investigated. In this research, we found that SGI-1027 not only suppressed the proliferation, migration and invasion of PTC cells, but also inhibited the glycolytic abilities of PTC cells both *in vitro* and *in vivo*. Thus, it indicated that we may provide the first evidence that SGI-1027 is an inhibitor of tumor growth and metastasis for PTC by affecting the dynamic characteristics of regulating the methylation status of some crucial genes. Utilization of SGI-1027 might be a promising strategy against the growth and metastasis of PTC.

## Supplementary Material

Supplementary figures and tables.

## Figures and Tables

**Table 1:** Methylation modification sites in the upstream of FAM111B

Methylation sites	Correlation	P value
cg20107987	0.086	<0.05
cg21833459	-0.014	>0.05
cg03004497	-0.053	>0.05
cg09583654	-0.042	>0.05
cg02404739	-0.111	<0.01
cg23633158	0.02	>0.05
cg11139304	0	null
cg16540026	0	null
cg05172141	0	null
cg14859464	-0.252	<0.001
cg13864937	-0.121	<0.01
cg13912641	-0.078	>0.05
cg10011647	0.023	>0.05
