# Supplementary material for: DNMT3B-mediated FAM111B methylation promotes papillary thyroid tumor glycolysis, growth and metastasis
Source: Int J Biol Sci. 2022 Jul 4;18(11):4372–87. doi: 10.7150/ijbs.72397 (PMC9295055; doi:10.7150/ijbs.72397)
Supplement: Supplementary file 1 — Supplementary figures and tables. [file ijbsv18p4372s1.pdf]

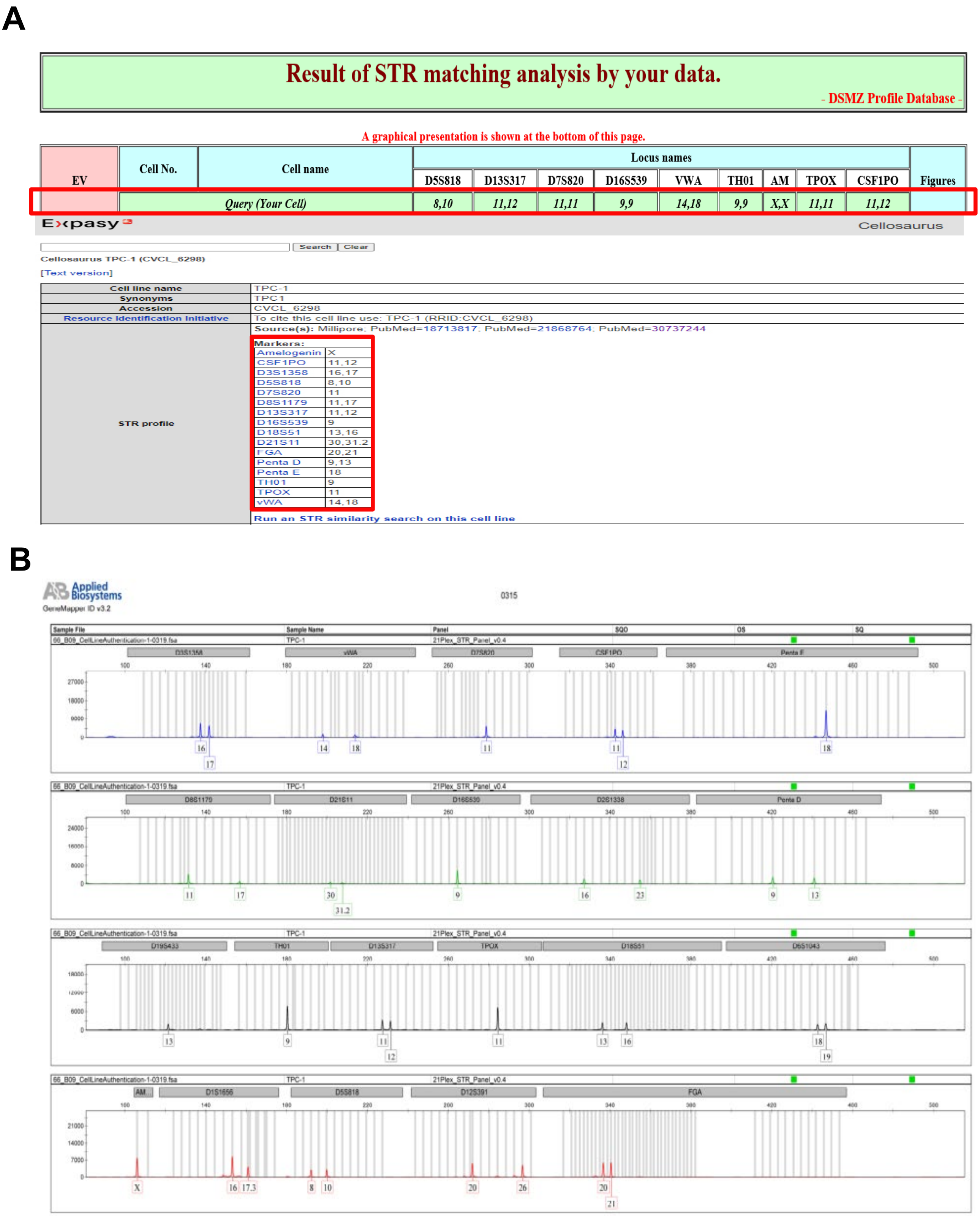

A

Result of STR matching analysis by your data.

- DSMZ Profile Database -

A graphical presentation is shown at the bottom of this page.

EV	Cell No.	Cell name	D5S818	D13S317	D7S820	D16S539	VWA	TH01	AM	TPOX	CSF1PO	Figures
		Query (Your Cell)	*8,10*	*11,12*	*11,11*	*9,9*	*14,18*	*9,9*	*X,X*	*11,11*	*11,12*	

Expasy — Cellosaurus

Search Clear

Cellosaurus TPC-1 (CVCL_6298)

[Text version]

Field	Value
Cell line name	TPC-1
Synonyms	TPC1
Accession	CVCL_6298
Resource Identification Initiative	To cite this cell line use: TPC-1 (RRID:CVCL_6298)
STR profile	Source(s): Millipore; PubMed=18713817; PubMed=21868764; PubMed=30737244

Markers:

Marker	Alleles
Amelogenin	X
CSF1PO	11,12
D3S1358	16,17
D5S818	8,10
D7S820	11
D8S1179	11,17
D13S317	11,12
D16S539	9
D18S51	13,16
D21S11	30,31.2
FGA	20,21
Penta D	9,13
Penta E	18
TH01	9
TPOX	11
vWA	14,18

Run an STR similarity search on this cell line

B

Supplementary Figure 1 Authentication of TPC-1 cell line we used by STR profile.
(A) Searching for the matching results of locus for TPC-1 cell line we used in the DSMZ profile and Cellosaurus database (in red frame). (B) Sequencing for locus of TPC-1 cell line we used and presentation of each characteristic locus.

A

Result of STR matching analysis by your data.

- DSMZ Profile Database -

A graphical presentation is shown at the bottom of this page.

EV	Cell No.	Cell name	Locus names									Figures
			D5S818	D13S317	D7S820	D16S539	VWA	TH01	AM	TPOX	CSF1PO	
	Query (Your Cell)		*11,11*	*12,12*	*10,10*	*11,12*	*14,17*	*6,9.3*	*x,x*	*8,11*	*13,13*	
0.94(34/36)	273	B-CPAP	10,11	12,12	10,10	11,12	14,17	6,9.3	X,X	8,11	13,13	-
0.70(26/37)	CRL-5803	NCI-H1299	11,11	12,12	10,10	12,13	16,17,18	6,9.3	X,X	8,8	12,12	-
0.67(24/36)	266	S-117	11,11	12,12	11,11	11,14	14,14	6,6	X,X	8,8	13,13	-
0.67(24/36)	446	CAL-27	11,12	10,11	10,10	11,12	14,17	6,9.3	X,X	8,8	10,12	-
0.67(24/36)	572	SU-DHL-6	12,12	12,14	10,10	11,12	14,17	6,9.3	X,X	11,12	10,10	-
0.67(24/36)	687	MINO	11,12	12,12	10,11	11,12	14,17	9.3,9.3	X,Y	8,11	9,11	-
0.67(24/36)	766	PGA-1	11,12	8,12	10,11	12,12	17,17	6,9.3	X,Y	8,11	13,13	-
0.67(24/36)	768	U-2904	11,11	12,12	12,12	11,13	16,17	6,9.3	X,X	8,11	12,14	-
0.67(24/36)	776	PG-EBV	11,12	8,12	10,11	9,12	17,17	6,9.3	X,Y	8,11	13,13	-
0.67(24/36)	781	HS-578T	11,11	11,11	10,10	12,12	17,17	9,9.3	X,X	8,8	13,13	-
0.67(24/36)	CCL-251	NCI-H716 [H716]	11,11	8,11	10,11	11,12	16,17	6,9.3	X,X	8,11	11,11	-
0.67(24/36)	CRL-1486	HEPM	11,13	8,12	8,10	11,12	17,18	6,9.3	X,X	8,11	10,11	-
0.67(24/36)	CRL-2095	CAL 27	11,12	10,11	10,10	11,12	14,17	6,9.3	X,X	8,8	10,12	-

B

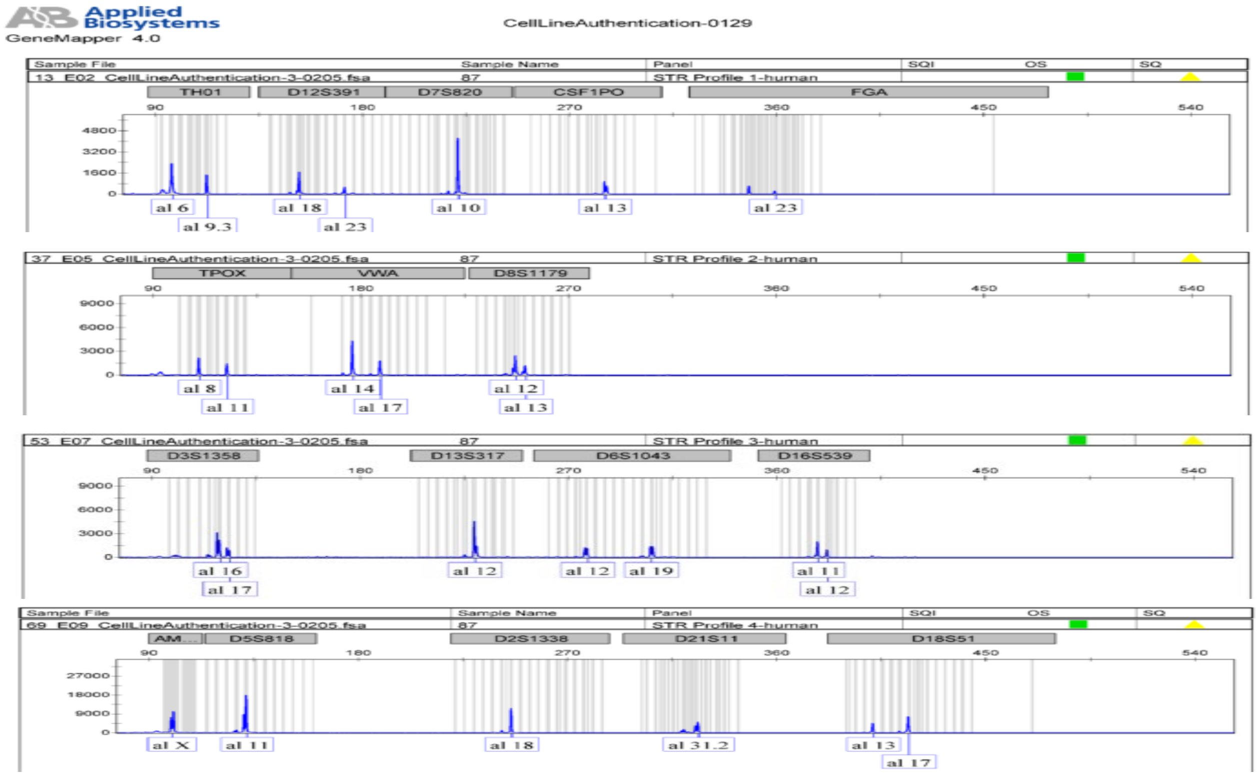

Supplementary Figure 2 Authentication of B-CPAP cell line we used by STR profile.
(A) Searching for the matching results of locus for B-CPAP cell line we used in the DSMZ profile database (in red frame).
(B) Sequencing for locus of B-CPAP cell line we used and presentation of each characteristic locus.

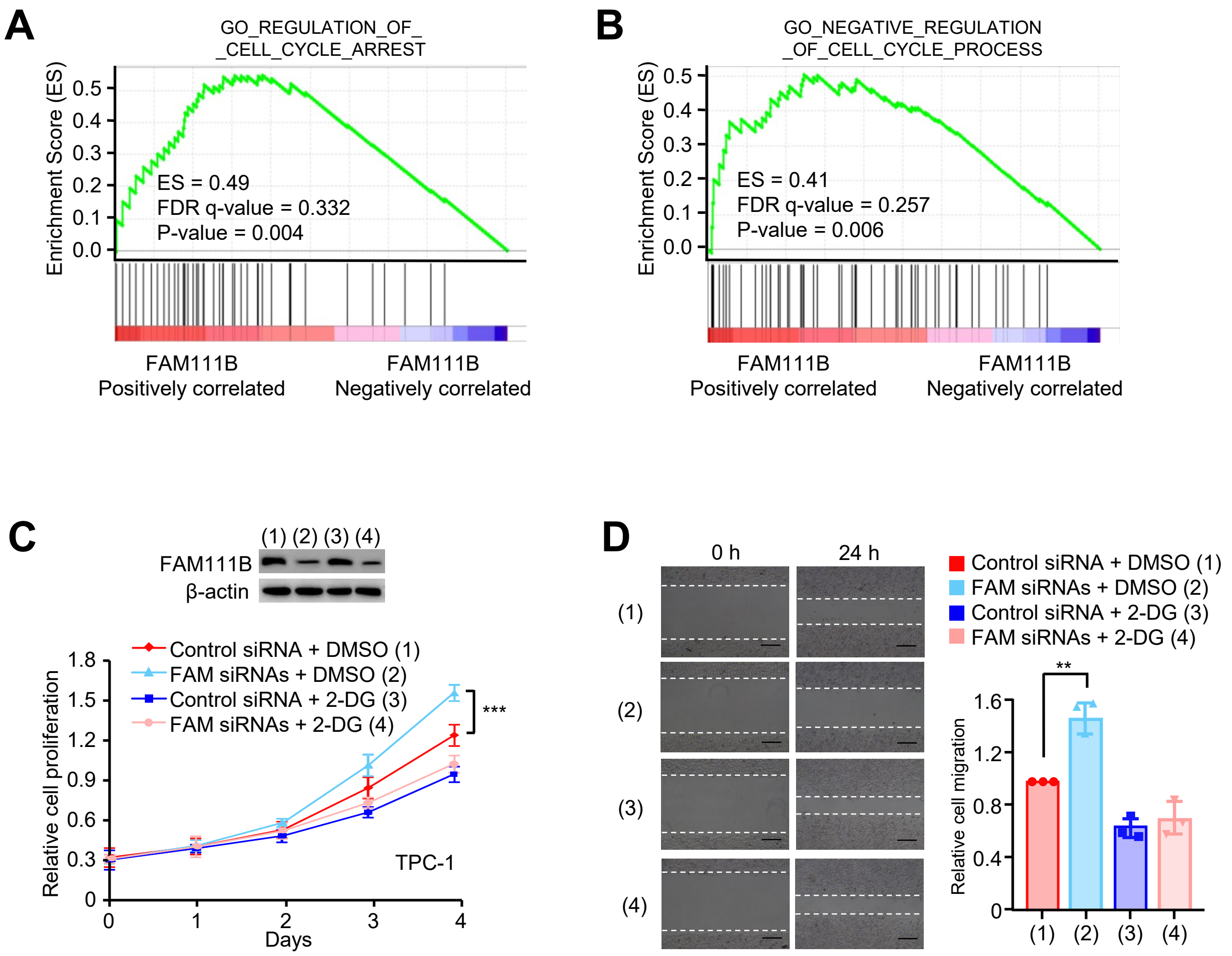

Supplementary Figure 3 2-DG inhibits the growth and migration of PTC.
(A, B) GSEA plot showing that FAM111B expression is positively correlated with cell cycle arrest and negetively correlated with cell cycle process in the TCGA THCA dataset. (C) TPC-1 cells were transfected with FAM111B siRNAs and treated with 2-DG. The proliferation of the cells was measured by CCK-8 assay. (D) Wound healing of TPC-1 cells transfected and treated as in (C). Right histograms reveal the relative cell migration. Scale bar, 50 μm. ***p* < 0.01, ****p* < 0.001 vs. Control siRNA + DMSO group

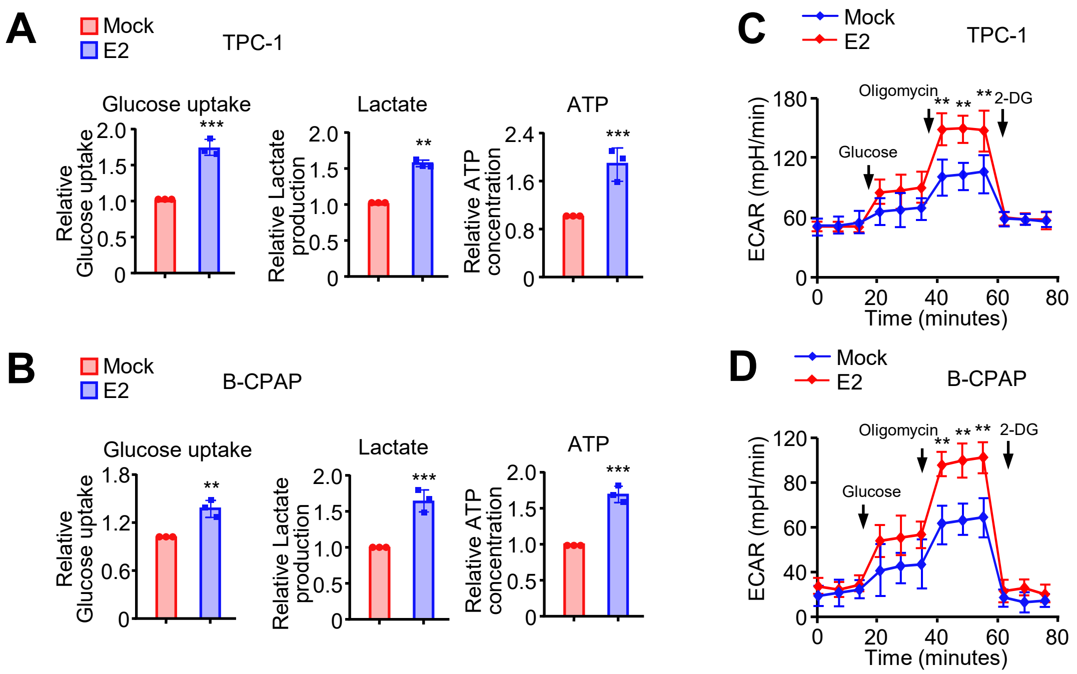

Supplementary Figure 4 E2 upregulates glycolysis in PTC cells.

(A, B) TPC-1 and B-CPAP cells were treated with Mock or E2 respectively. Glucose uptake, lactate production and ATP production were measured. (C, D) TPC-1 cells (C) and B-CPAP cells (D) were treated as in (A,B), and extracellular acidification rate (ECAR) was then evaluated. The arrows indicate the time of adding glucose, oligomycin, 2-DG. All values shown are mean ± S.D. of triplicate measurements and have been repeated 3 times with similar results (A-D). ***p* < 0.01, ****p* < 0.001 vs. Mock group.

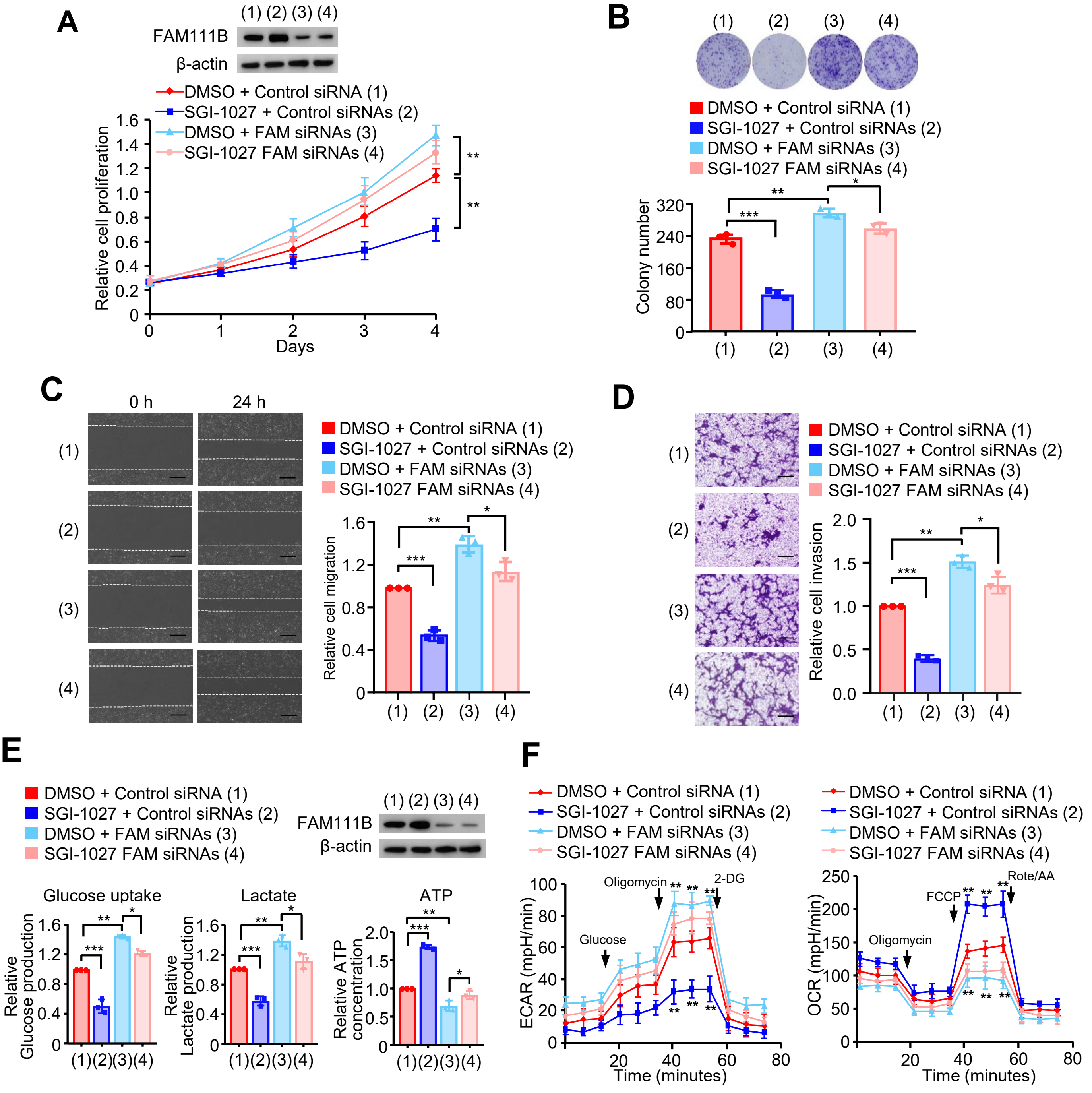

Supplementary Figure 5 Methylation of FAM111B by DNMT3B promotes the growth, migration, invasion and glycolysis of PTC. (A) B-CPAP cells were transfected with FAM111B siRNAs and treated with SGI-1027. The proliferation of the cells was measured by CCK-8 assay. The representative immunoblot indicated FAM111B protein level. (B) Colony formation assay of B-CPAP cells transfected and treated as in (A). Representative images reveal colonies in plates (upper panels). Histograms reveal colony number. (C, D) Wound healing (C) and Transwell (D) assays of B-CPAP cells transfected and treated as in (A). Right histograms reveal the relative cell migration and invasion. (E) Glucose uptake and the production of lactate and ATP were determined. Representative immunoblot shows the expression of FAM111B. (F) B-CPAP cells were transfected and treated as in (A), and extracellular acidification rate (ECAR) (F) and oxygen consumption rate (OCR) were then measured. Scale bar, 50 μm. *$p < 0.05$, **$p < 0.01$, ***$p < 0.001$ vs. DMSO + Control siRNA group.

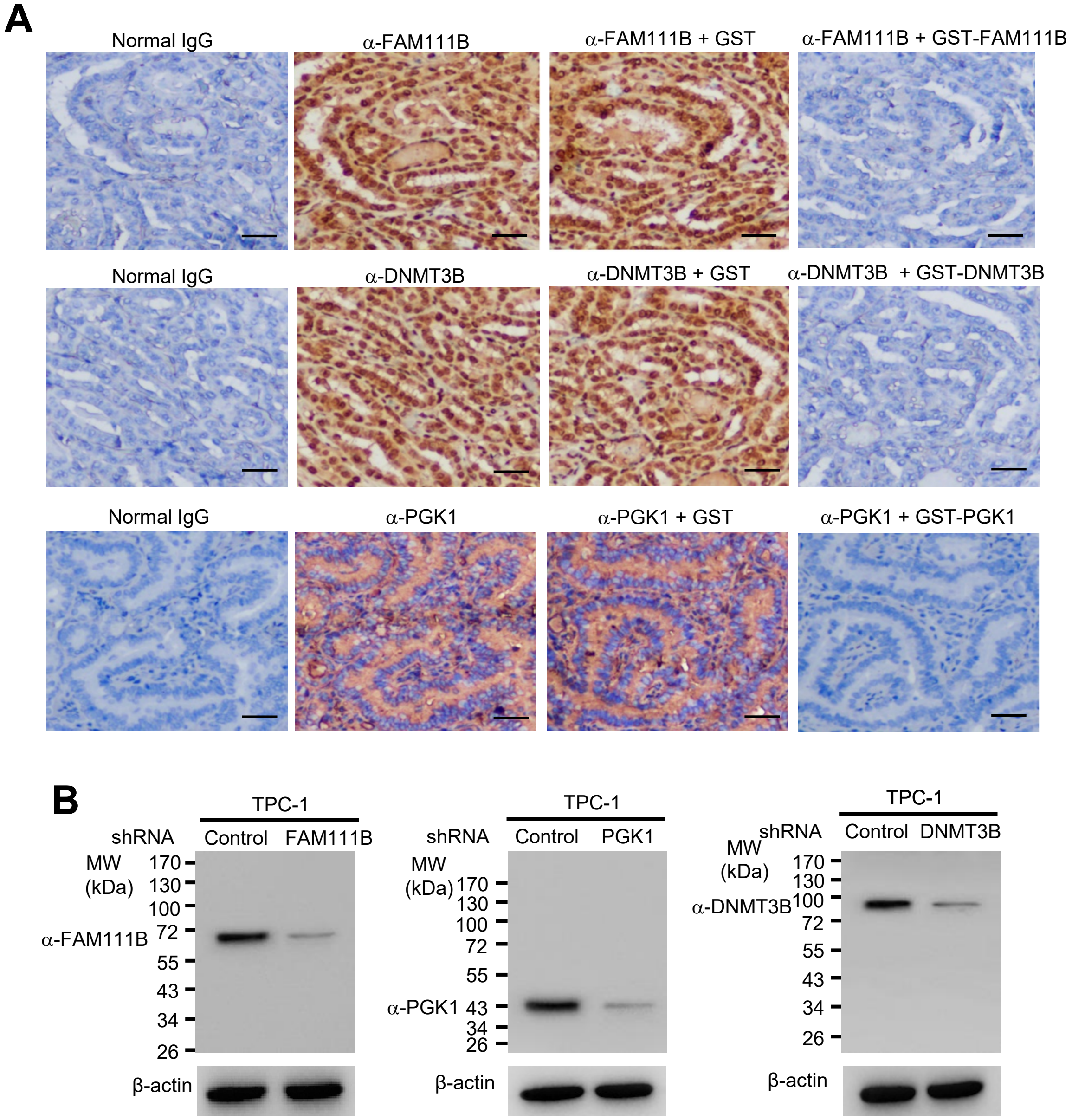

Supplementary Figure 6 Identification of the specificity of the antibodies used in IHC.
(A) IHC staining of thyroid cancer specimens incubated with normal IgG, anti-FAM111B, anti-DNMT3B and anti-PGK1. To confirm the antibody specificity, anti-FAM111B, anti-DNMT3B and anti-PGK1 was pre-incubated respectively with the indicated recombinant GST or GST-FAM111B, GST-DNMT3B and GST-PGK1 applied to the tissue for 1 hour. Scale bar, 50 μm. (B) Western blot analysis of lysates from TPC-1 cells infected with control shRNA or FAM111B, PGK1 and DNMT3B.

Table S1 The sequences of all primers and oligonucleotide used in the study.

Gene	Forward primer (5' to 3')	Reverse primer (5' to 3')
FAM111B	GCCCTTGAAATGCAGAATCCA	GCTGTAAACACACTACGGTCTAA
ALDOA	ATGCCCTACCAATATCCAGCA	GCTCCCAGTGGACTCATCTG
DNMT3B	AGGGAAGACTCGATCCTCGTC	GTGTGTAGCTTAGCAGACTGG
β-ACTIN	GGGACCTGACTGACTACCTC	TCATACTCCTGCTTGCTGAT
GPI	CAAGGACCGCTTCAACCACTT	CCAGGATGGGTGTGTTTGACC
PGAM4	CCTCAGTGCAGAAGAGAGTGATC	TGGTGGGACATCATAGGAGCGC
PGAM2	AGAAGCACCCCTACTACAACTC	TCTGGGGAACAATCTCCTCGT
PGAM1	GTGCAGAAGAGAGCGATCCG	CGGTTAGACCCCCATAGTGC
LDHA	ATGGCAACTCTAAAGGATCAGC	CCAACCCCAACAACTGTAATCT
PFKP	GCATGGGTATCTACGTGGGG	CTCTGCGATGTTTGAGCCTC
PFKL	GCTGGGCGGCACTATCATT	TCAGGTGCGAGTAGGTCCG
ENO1	AAAGCTGGTGCCGTTGAGAA	GGTTGTGGTAAACCTCTGCTC
PGM2	GAGGCAGTGAAACGACTAATAGC	CTGTCCCAAACTCCATTCGGG
PGM1	CCAAACCGACTGAAGATCCGT	CATGTTTCGATCCCCATCTCC
GAPDH	GGAGCGAGATCCCTCCAAAAT	GGCTGTTGTCATACTTCTCATGG
PGK1	TGGACGTTAAAGGGAAGCGG	GCTCATAAGGACTACCGACTTGG
HK2	GAGCCACCACTCACCCTACT	CCAGGCATTCGGCAATGTG
PFKM	GGTGCCCGTGTCTTCTTTGT	AAGCATCATCGAAACGCTCTC
ALDH2	ATGGCAAGCCCTATGTCATCT	CCGTGGTACTTATCAGCCCA
PKM	GACCCGGAATCCCCAGACAG	TCACGGCACAGGAACAACACG
**Oligonucleotide**	**Sense (5' to 3')**	**Antisense (5' to 3')**
si-FAM111B -1	CCUGUUGAUCAUUGUCUAUTT	AUAGACAAUGAUCAACAGGTT
si-FAM111B-2	GCAAGUCAGUUGGGUUCAUTT	AUGAACCCAACUGACUUGCTT
si-FAM111B-3	GCAUGCCCUUAUUGAAUUUTT	AAAUUCAAUAAGGGCAUGCTT

Table S2 The sequences of FAM111B-R

ATGAATTCCATGAAGACTGAAGAAAACAAGTCATTTAGCGCTATGGAAGATGACCAGA<br>GGACTAGACCTGAAGTTTCAAAGGATACTGTCATGAAGCAGACACATGCTGACACACC<br>TGTTGATCACTGTCTATCTGGCATAAGAAAGTGTAGCAGCACCTTTAAGCTTAAAAGTG<br>AAGTCAACAAGCATGAAACAGCCCTTGAAATGCAGAATCCAAATTTGAACAATAAAGA<br>ATGTTGTTTCACCTTTACGTTGAATGGAAACTCCAGAAAATTAGACCGTAGTGTGTTTA<br>CAGCATATGGTAAACCCAGCGAGAGTATCTACTCAGCCCTGAGTGCTAATGACTATTTC<br>AGTGAAAGGATAAAGAATCAGTTTAATAAGAACATTATTGTTTATGAAGAAAAGACAAT<br>AGATGGACATATAAATTTAGGAATGCCTCTCAAGTGCCTGCCTAGTGATTCTCATTTTAA<br>AATTACATTTGGTCAAAGAAAGAGTAGCAAAGAAGATGGACACATATTACGCCAATGT<br>GAAAATCCAAACATGGAATGCATTCTTTTTCATGTTGTTGCTATAGGAAGGACAAGAAA<br>GAAGATTGTTAAGATCAACGAACTTCATGAAAAAGGAAGTAAACTTTGTATTTATGCCT<br>TGAAGGGTGAGACTATTGAAGGAGCCTTATGCAAGGATGGCCGTTTTCGGTCTGACAT<br>AGGTGAATTTGAATGGAAACTAAAGGAAGGTCATAAGAAAATTTATGGAAAACAGTCC<br>ATGGTGGATGAAGTATCTGGAAAAGTCTTAGAAATGGACATTTCAAAAAAAAAAGCAT<br>TACAACAGAAAGATATCCATAAAAAAATTAAACAGAATGAAAGTGCCACTGATGAAAT<br>TAATCACCAGAGTCTGATACAGTCTAAGAAAAAAGTCCACAAACCAAAGAAAGATGG<br>AGAGACCAAAGATGTAGAACACAGCAGAGAGCAAATTCTCCCACCTCAGGATCTAAG<br>CCATTATATTAAAGATAAAACTCGCCAGACAATTCCCAGGATTAGAAATTATTACTTTTG<br>TAGTTTGCCCCGAAAATATAGGCAAATAAACTCACAAGTTAGACGGAGGCCGCATCTG<br>GGTAGGCGGTATGCTATTAATCTGGATGTCCAAAAGGAGGCAATTAATCTCTTAAAGAA<br>TTATCAAACGTTGAATGAAGCCATAATGCATCAGTATCCGAATTTTAAAGAGGAGGCAC<br>AGTGGGTAAGAAAATATTTTCGGGAAGAACAAAAGAGAATGAATCTTTCACCAGCTAA<br>GCAATTCAACATATATAAAAAGGACTTCGGAAAAATGACTGCAAATTCTGTTTCAGTTG<br>CAACCTGCGAACAGCTTACATATTATAGCAAGTCAGTCGGGTTCATGCAATGGGACAAT<br>AATGGAAACACAGGTAATGCTACTTGCTTTGTCTTCAATGGTGGTTATATTTTCACCTGT<br>CGACATGTTGTACATCTTATGGTGGGTAAAAACACACATCCAAGTTTGTGGCCAGATAT<br>AATTAGCAAATGTGCGAAGGTAACCTTCACTTATACAGAGTTCTGCCCTACTCCTGACA<br>ATTGGTTTTCCATTGAGCCATGGCTTAAAGTGTCCAATGAAAATCTAGATTATGCCATTT<br>TAAAACTAAAAGAAAATGGAAATGCGTTTCCTCCAGGACTATGGCGACAGATTTCTCC<br>TCAACCATCTACTGGTTTGATTTATTTAATTGGTCATCCTGAAGGCCAGATCAAGAAAAT<br>AGATGGTTGTACTGTGATTCCTCTAAACGAACGATTGAAAAAATATCCAAACGATTGTC<br>AAGATGGGTTGGTAGATCTCTATGATACCACCAGTAATGTATACTGTATGTTTACCCAAA<br>GAAGTTTCCTATCAGAGGTTTGGAACACACACACGCTTAGTTATGATACTTGTTTCTCT<br>GATGGGTCCTCAGGCTCCCCAGTGTTTAATGCATCTGGCAAATTGGTTGCTTTGCATAC<br>CTTTGGGCTTTTTTATCAACGAGGATTTAATGTGCATGCCCTCATTGAATTTGGTTATTCT<br>ATGGATTCTATTCTTTGTGATATTAAAAAGACAAATGAGAGCTTGTATAAATCATTAAAT<br>GATGAGAAACTTGAGACCTACGATGAAGAGAAAGGTAAACAAGAGTCATCACTTCAA<br>GATCATCAGATTGAACCCATGGAATGTTAG

The red font represents the target sequence of siRNAs and the yellow font represents the mutation sites.
